# Red and Blue Light Promote the Accumulation of Artemisinin in *Artemisia annua* L.

**DOI:** 10.3390/molecules23061329

**Published:** 2018-05-31

**Authors:** Dong Zhang, Wei Sun, Yuhua Shi, Lan Wu, Tianyuan Zhang, Li Xiang

**Affiliations:** 1 Artemisinin Reserch Center, China Academy of Chinese Medical Sciences, Beijing 100700, China; dzhang1987@icmm.ac.cn (D.Z.); yhshi@icmm.ac.cn (Y.S.); lwu@icmm.ac.cn (L.W.); 2 Institute of Chinese Materia Medica, China Academy of Chinese Medical Sciences, Beijing 100700, China; wsun@icmm.ac.cn (W.S.); zhangtianyuan@foxmail.com (T.Z.)

**Keywords:** *Artemisia annua*, artemisinin, RNA sequencing (RNA-seq), light, secondary metabolism

## Abstract

Artemisinin, which has been isolated from *Artemisia annua* L., is the most effective antimalarial drug and has saved millions of lives. In addition, artemisinin and its derivatives have anti-tumor, anti-parasitic, anti-fibrosis, and anti-arrhythmic properties, which enhances the demand for these compounds. Improving the content of artemisinin in *A. annua* is therefore becoming an increasing research interest, as the chemical synthesis of this metabolite is not viable. Ultraviolet B and C irradiation have been reported to improve the artemisinin content in *A. annua*, but they are harmful to plant growth and development. Therefore, we screened other light sources to examine if they could promote artemisinin content without affecting plant growth and development. We found that red and blue light could enhance artemisinin accumulation by promoting the expression of the genes that were involved in artemisinin biosynthesis, such as *amorpha-4,11-diene synthase* (ADS) and *cytochrome P450 monooxygenase* (CYP71AV1) genes. Thus, in addition to being the main light sources for photosynthesis, red and blue light play a key role in plant secondary metabolism, and optimizing the combination of these light might allow for the productionof artemisinin-rich *A. annua*.

## 1. Introduction

Malaria, a serious disease that is caused by *Plasmodium* sp. infection, threatens human health and safety in 108 countries and regions. About 3300 million people are exposed to malaria, more than 100 million are infected, and nearly 800,000 die every year. The World Health Organization ranks it alongside acquired immune deficiency syndrome (AIDS) and tuberculosis as one of the world's top three public health problems [1]. Artemisinin-based therapies are the main and best treatments for malaria [2], and the discovery of artemisinin as well as its contribution to malaria treatment granted Professor Youyou Tu the 2015 Nobel Prize in Physiology or Medicine. Artemisinin and its derivatives have also manifested anti-tumor, anti-parasitic, anti-fibrosis, and anti-arrhythmic properties [3]. Therefore, an increasing demand for artemisinin and its derivatives is expected [4]. Presently, the main source of artemisinin is through direct extraction from *Artemisia annua* L. Although artemisinin has been chemically synthesized since 1983, this method has complex steps and thus is far from industrialization, as is the synthesis of artemisinin by cell culture. In addition, artemisinin can be obtained by heterologous synthesis in *Escherichia coli* or yeast [5]. Because the artemisinin content is very low in most varieties of *A. annua*, improving its content in this plant is an urgent requirement.

Plant secondary metabolites are greatly affected by various environmental factors, such as light, temperature, humidity, and pathogenic bacteria [6,7,8]. Generally, plants under biotic or abiotic stress produce a large number of secondary metabolites as a defense against such stress. Light is an important environmental factor that regulates a plant’s life cycle, including plant growth and development [9,10,11]. Several studies have reported that ultraviolet (UV)-B irradiation can promote the accumulation of flavonoids in buckwheat [12,13], by enhancing the expression of key metabolic genes, such as *phe ammonia lyase* (PAL), *cinnamate-4-hydroxylase* (C4H), *chalcone synthase* (CHS), *chalcone isomerase* (CHI), and *flavonol synthase* (FLS). In addition, resveratrol accumulation in mature grape berries is induced by UV-C irradiation [14,15,16,17]. It was also found that UV-B and -C irradiation could improve the content of artemisinin in *A. annua*. In the plants that were treated with UV-B and -C irradiation for a short time (14 days) before flowering, the artemisinin content increased by 1.63 and 1.75 times, respectively, compared with the control group [18].

**Figure 1:**
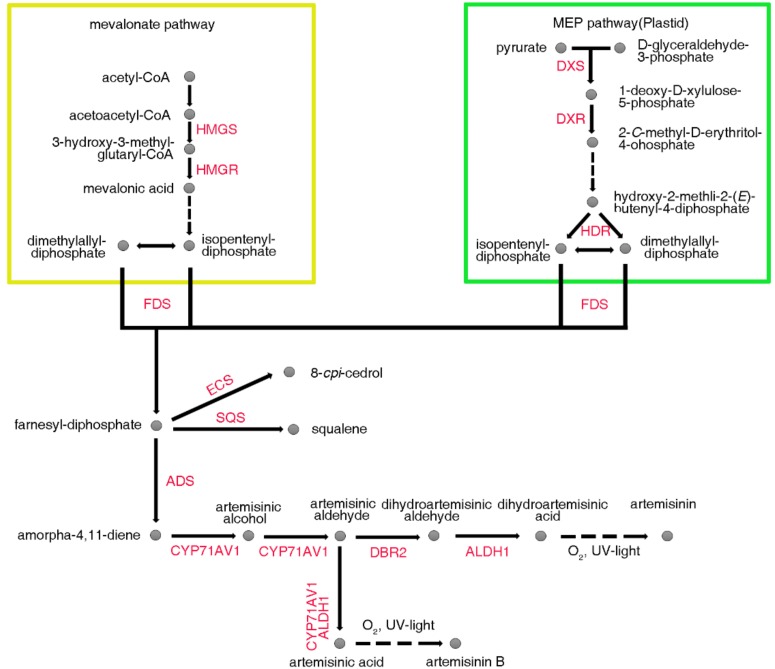
Schematic representation of artemisinin biosynthesis in *Artemisia annua* L. HMGS—3-hydroxy-3-methyl-glutaryl coenzyme A synthase; HMGR—3-hydroxy-3-methyl-glutaryl coenzyme A reductase; DXS—1-deoxy-d-xylulose-5-phosphate synthase; DXR—1-deoxy-Dxylulose-5-phosphate reductoisomerase; HDR—hydroxy-2-methyl-2-(*E*)-butenyl 4-diphosphate reductase; FDS—farnesyl diphosphate synthase; SQS—squalene synthase; ECS—epi-cedrol synthase; ADS—amorpha-4,11-diene synthase; CYP71AV1—amorphadiene-12-hydroxylase; DBR2—artemisinic aldehyde Δ11(13) reductase; ALDH1—aldehyde dehydrogenase 1; MEP—methylerythritol phosphate.

Artemisinin is a sesquiterpene lactone with a peroxy bridge structure (i.e., 1,2,4-three dioxane ring) and its biosynthetic pathway has been clearly identified (Figure 1). In plants, this five carbon-skeleton compound has two independent biosynthetic pathways, which are the mevalonate (MVA) pathway and are located in the cytoplasm, and the methylerythritol phosphate (MEP) pathway, which occur in plastids [19]. The metabolic steps before the farnesyl diphosphate (FPP) synthesis are considered as an upstream of artemisinin biosynthesis, and FPP is formed by isopentenyl diphosphate (IPP), which is provided by the MVA and MEP pathways [19]. Under the catalysis of amorpha-4,11-diene synthase (ADS), FPP closed-loop produces amorpha-4,11-diene [20], which is oxidized to sequentially generate artenimol, artemisinic aldehyde, and artemisinic acid, which is catalyzed by the cytochrome P450 monooxygenase CYP71AV1 [21,22,23]. Under the catalysis of artemisinic aldehyde delta11(13)-reductase (DBR2) and aldehyde dehydrogenase 1 (ALDH1), artemisinic aldehyde is converted to dihydroartemisinic acid [24], which is converted to artemisinin.

Over the past decades, the biosynthetic pathway of artemisinin has been clearly understood. However, the transcriptional regulation of artemisinin is relatively slow and the synthesis and accumulation of secondary metabolites have specific temporal and spatial patterns [25]. Transcription factors (TFs), which are the proteins that bind to specific sequences of downstream target genes, and that activate or inhibit gene expression, play an important role in this type of regulation [26] and, in recent years, the discovery of some TFs has promoted the study of artemisinin transcriptional regulation [27,28,29,30,31]. Therefore, studying their function is crucial for understanding artemisinin biosynthesis and its regulation. It has been found that WRKY (named after its WRKY domain, which binds to the DNA sequence), APETALA2/ethylene-responsive (AP2/ERF), basic helix–loop–helix (bHLH), and basic leucine zipper (bZIP) are involved in the regulation of artemisinin biosynthesis. Ma et al. [27] cloned AaWRKY1, which can promote the expression of ADS in *A. annua.* Lu et al. [28] cloned the AP2/ERF TF AaORA, which was specifically expressed in *A. annua* trichomes, and by overexpressing the AaORA gene, this also promoted the overexpression of the artemisinin biosynthetic pathway genes ADS, CYP71AV1, and DBR2, resulting in an increase of artemisinin contents by 40–53% in overexpressing transgenic plants. Ji et al. [29] cloned AabHLH1, which also can promote the expression of artemisinin biosynthesis genes ADS and CYP71AV1. Recently, the bHLH TF AaMYC2, which was cloned by Tang et al. [30], was found to promote the expression of CYP71AV1 and DBR2 genes. Zhang et al. [31] found that the TF AabZIP1, which mediates abscisic acid (ABA) signaling, was able to promote the synthesis of artemisinin by promoting the expression of ADS and CYP71AV1 genes. Compared with the wild-type, the artemisinin content was increased from 0.7- to 1.5-fold in AabZIP1-OX lines.

The present study comprehensively evaluated the impact of different light waves (far-red, white, blue, and red) on the synthesis of artemisinin. Using transcriptome analysis, several candidate genes that transcriptionally regulate the synthesis of artemisinin were found, providing important information for further research regarding the regulation of artemisinin synthesis. The use of other light sources to promote the content of artemisinin not affecting the growth and development of *A. annua* have been discussed regarding the establishment of *A. annua* plantations for artemisinin intensive production.

## 2. Results and Discussion

### 2.1. Analysis of Artemisinin Content under Various Treatments

**Figure 2:**
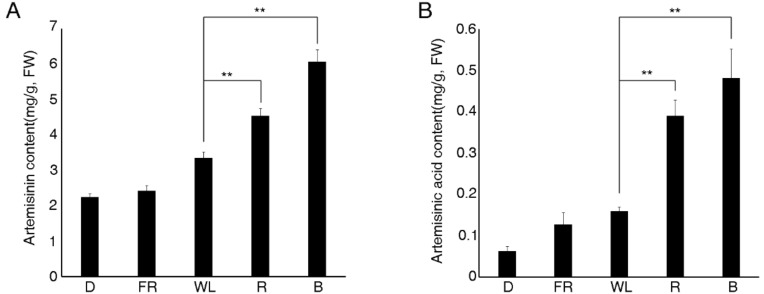
Artemisinin (**A**) and artemisinic acid (**B**) content under different light treatments. In (**A**) and (**B**), data represent means ± standard deviation of biological triplicates (10 seedlings per repeat). D—darkness; FR—far red light; WL—white light; R—red light; B—blue light; FW—fresh weight. Double asterisks indicate significant differences at *p* < 0.01 using Student’s *t*-test.

The internal transcribed spacer 2 (ITS2) ribosomal DNA sequence was characterized by high nucleotide variation and thus was widely used in phylogenetic and taxonomic studies. The ITS2 sequences of the samples that were collected in Hainan, China, for the purposes of the present study, completely matched the ITS2 sequence of *A. annua* (data not shown), which confirmed the identity of the collected plants. After sowing the *A. annua* seeds into the soil for four weeks under white light, the seedlings were transferred to darkness, white light, far-red light, red light, and blue light conditions for two days. After the light treatments, the chlorophyll content was detected, and the seedlings under red and blue light accumulated increased levels of chlorophyll compared with the white light treatment (Figure S1). In addition, the artemisinin and artemisinic acid content was analyzed by liquid chromatography–mass spectrometry (LC–MS). The results showed that light had an important effect on the content of artemisinin and artemisinic acid, as blue and red light notably increased the content of these compounds, compared with white light. Under far-red light and darkness conditions, the content of artemisinin was equal to and obviously lower than under the white light, respectively (Figure 2). Overall, these results indicated that light, especially blue and red light, could enhance the content of artemisinin and artemisinic acid.

Light not only provided energy for plant photosynthesis, but it also acted as an important environmental signal so as to regulate several types of plant growth and development processes, such as the transition from heterotrophic to autotrophic growth, which involves chlorophyll synthesis, chloroplast development, and photomorphogenesis [9,10,11]. However, light could be a stress factor regulating plant secondary metabolism. Although UV-B and -C light could improve the content of artemisinin in *A. annua* [18], UV light was harmful to organisms, causing DNA mutations. Thus, examining the effects of other light on the synthesis of artemisinin was crucial for cultivating *A. annua* plants that were able to produce high amounts of this metabolic compound. In plants, there were several important light receptors, including phytochromes, which perceived far-red and red light signals [32]; cryptochromes, which perceived blue light signals [33,34,35]; and UVR8, which perceived UV-B signals [36]. Far-red, red, and blue light were used to treat the *A. annua* seedlings. Our results showed that blue and red light notably increased the content of artemisinin compared with the white light (Figure 1). This was of great significance, as red and blue light did not harm plant growth [37].

### 2.2. Transcriptome Sequencing and Assembly

The samples were subjected to Illumina Xten paired-end sequencing. After removing the adaptor sequences and filtering the low quality and ambiguous reads, 713 million clean reads that contained 107 Gb of valid data, were acquired. A summary of the sequencing statistics is shown in Table 1. These reads were assembled into 651,000 transcripts and 271,932 unigenes using Trinity. The average length of the transcripts was 922 bp and the shortest sequence length, at 50% of the genome (N50), was 1501 bp. The average length of the unigenes was 711 bp and that of N50 was 1176 bp. The maximum length of both transcripts and unigenes was 18,284 bp, and their minimum length was 201 bp. However, the transcripts had 600,292,436 nucleotides in total, whereas the unigenes had only 193,418,027 nucleotides. The sequencing data have been deposited in the National Center for Biotechnology Information (NCBI) database (Accession number: SRP133983)

### 2.3. Functional Annotation and Classification

For functional annotation, all of the assembled unigenes were searched against the NCBI non-redundant protein database (NR), NCBI nucleotide sequences (NT), Swiss-Prot, Protein family (PFAM), euKaryotic Ortholog Groups (KOG), gene ontology (GO), and Kyoto Encyclopedia of Genes and Genomes (KEGG) databases (Table 2).

**Figure 3:**
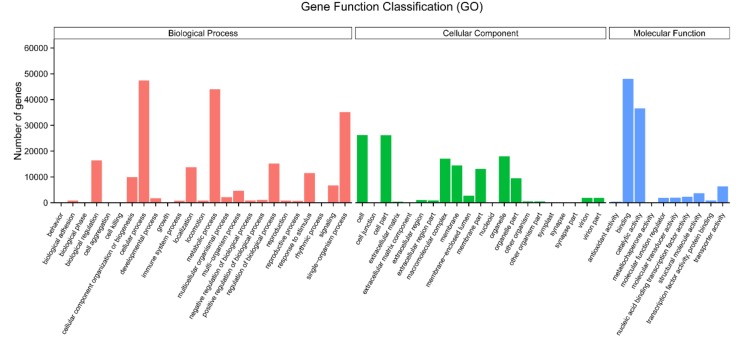
Functional annotation of all of the assembled unigenes into biological process, cellular component, and molecular function categories within the gene ontology (GO) database.

For the global functional analysis, 87,236 unigenes were annotated in the GO database and were classified into 56 functional groups, including 25 groups in the biological process, 21 in the cellular component, and 10 in the molecular function (Figure 3). Within the biological process, ‘cellular process’ (GO: 0009987; 47,389 unigenes) and ‘metabolic process’ (GO: 0008152; 43,972 unigenes) were predominant. In the cellular component category, the two main groups were the ‘cell’ (GO: 0005623; 26,159 unigenes) and ‘cell part’ (GO: 0044464; 26,129 unigenes). The terms ‘binding’ (GO: 0005488; 48,030 unigenes) and ‘catalytic activity’ (GO: 0003824; 36,550 unigenes) were the most common in the molecular function category. Comparing GO annotations of up-regulated genes under red and blue light treatments with those that were obtained under darkness, revealed that these genes were closely related to photosynthesis, which was consistent with previous reports stating that red and blue light were the main light sources for photosynthesis [37].

The KEGG pathway analysis, which was performed to identify metabolic pathways (Figure S2), revealed that 37,459 unigenes were grouped into 19 KEGG pathways, mainly including translation (5046), metabolism (4818), carbohydrate metabolism (3414), as well as folding, sorting, and degradation (3176).

The KOG pathway analysis (Figure S3) revealed that 36,681 unigenes were grouped into 26 pathways, mainly including posttranslational modification, protein turnover, chaperones (5141), translation, ribosomal structure and biogenesis (4750), general function prediction only (4728), and signal transduction mechanisms (3611).

### 2.5. Comparative Analysis of Transcriptional Profiles

**Figure 4:**
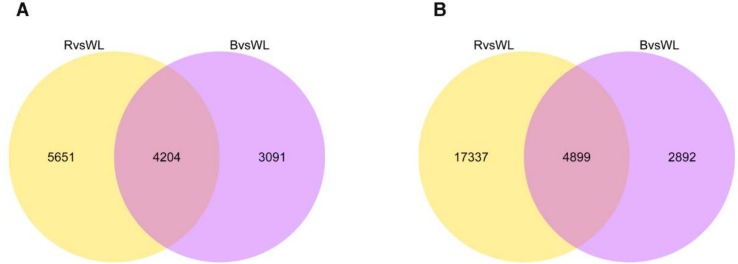
The Venn diagram shows the number of up-regulated (**A**) and down-regulated (**B**) unigenes in the two treatment groups. The numbers in the circles indicate the number of unigenes regulated by one or two treatment.

In order to identify differentially expressed genes (DEGs) in different treatment groups, the fragments per kilobase of exon per million mapped fragments (FPKM) values of the assembled unigenes were calculated. Under red light and blue light treatments, 9855 and 7295 unigenes, accounting for 3.6% and 2.7% of the total unigenes (271,932), respectively, were significantly overexpressed by at least 2-fold (Figure 4A). In addition, under red light and blue light, 22,236 and 7791 unigenes, accounting for 8.2% and 2.9% of the total unigenes, respectively, were significantly underexpressed by at least 2-fold (Figure 4B). The Venn diagram that was constructed for the number of up- and down-regulated unigenes among the red and blue light treatments displays overlapping and unique unigenes to each condition.

### 2.6. Expression Analysis of the Genes Involved in Artemisinin Biosynthesis

**Figure 5:**
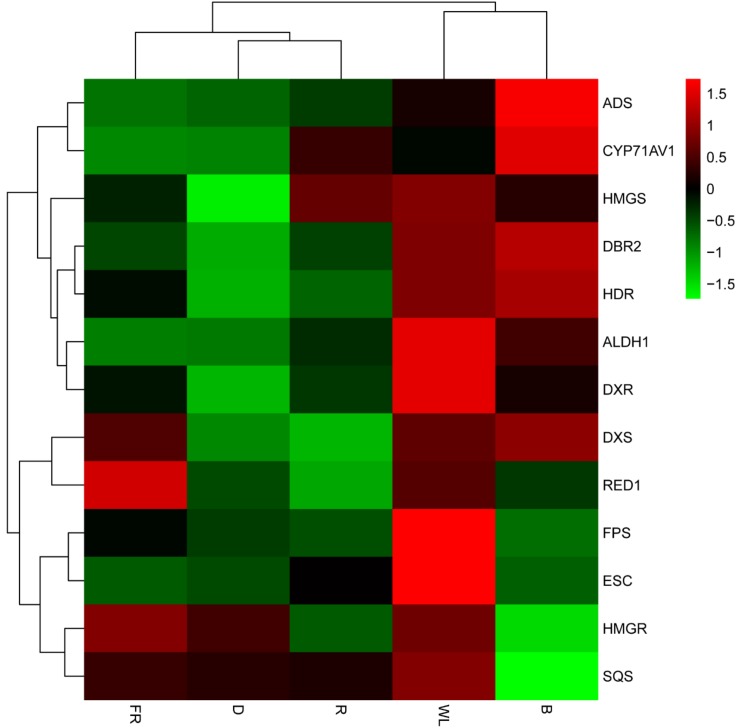
Heatmap showing variations in the expression of the genes involved in artemisinin biosynthesis. D—darkness; FR—far red light; WL—white light; R—red light; B—blue light. HMGS—3-hydroxy-3-methyl-glutaryl coenzyme A synthase; HMGR—3-hydroxy-3-methyl-glutaryl coenzyme A reductase; DXS—1-deoxy-d-xylulose-5-phosphate synthase; DXR—1-deoxy-Dxylulose-5-phosphate reductoisomerase; HDR—hydroxy-2-methyl-2-(*E*)-butenyl 4-diphosphate reductase; FPS—farnesyl diphosphate synthase; SQS—squalene synthase; ECS—epi-cedrol synthase; ADS—amorpha-4,11-diene synthase; CYP71AV1—amorphadiene-12-hydroxylase; DBR2—artemisinic aldehyde Δ11(13) reductase; ALDH1—aldehyde dehydrogenase 1. Color indicates the strength of the expression signal, with gene expression becoming stronger from green to red.

The transcriptome profiles revealed that many of the differentially expressed unigenes were annotated as artemisinin biosynthesis genes (Figure 5). In the mevalonate (MVA) pathway, two steps that were catalyzed by 3-hydroxy-3-methyl-glutaryl CoA synthase (HMGS) and 3-hydroxy-3-methyl-glutaryl CoA reductase (HMGR) converted acetoacetyl-CoA to mevalonic acid. This was reported when the overexpressed HMGR in *A. annua,* artemisinin content in the transgenic lines was 38.9% higher than that in non-transgenic plants [38]. In our results, the expression of HMGR was almost identical under dark or white light (control) conditions, which suggested that light could not promote the HMGR expression. However, the expression of HMGS was 3.1-fold higher under white light than in darkness, and red and blue light had almost the same effect as white light. 1-deoxy-d-xylulose-5-phosphate synthase (DXS) and 1-deoxy-d-xylulose-5-phosphate reductoisomerase (DXR), which were the first and second enzymes of the methylerythritol 4-phosphate (MEP) pathway and played a major role in the overall regulation of this route [39], catalyzed the conversion of pyruvate and d-glyceraldehyde-3-phosphate to 2-C-methyl-d-erythritol-4-phosphate, and hydroxy-2-methyl-2-(*E*)-butenyl 4-diphosphate reductase (HDR) catalyzed this compound to isopentenyl-diphosphate and dimethylallyl-diphosphate in darkness. Overexpression of HDR1 increased the contents of artemisinin, and the suppression of HDR1 led to opposite results [40]. The expression of DXS was slightly higher under white light than in darkness. However, the DXS and HDR expressions under white light were 3.3- and 2-fold higher than in darkness, respectively. Isopentenyl diphosphate and dimethylallyl diphosphate were utilized as substrates by FPS, to form FPP. Banyai et al. overexpressed FPS in *A. annua* and gained a 2.5-fold increase of artemisinin content at best [41]. Under white light, the expression of FPS was 2.5-fold higher than in darkness. Overall, the above results indicated that, by upstream FPP synthesis, light could have promoted the accumulation of artemisinin precursors and that white light was better than monochromatic light for enhancing such accumulation.

Downstream FPP synthesis, ADS, and CYP71AV1 were the most important enzymes. The content of artemisinin was increased by about 82% in the ADS overexpressing transgenic plant lines [27]. Jing et al. [42] overexpressed CYP71AV1 and CPR, and the artemisinin content was increased by as much as 2.4-fold compared with the control plant. Compared to darkness, the red light, white light, and blue light led to ADS overexpression by 2.5-, 5.9-, and 14.7-fold, respectively. Red light, white light, and blue light also led to the overexpression of CYP71AV1 by 24.3-, 16.7-, and 46.3-fold, respectively. Interestingly, the far-red light inhibited both of the enzymes, indicating that light promoted artemisinin synthesis in a complex process, as different light might have had different effects on the artemisinin biosynthesis gene expression. Blue light, in particular, promoted the expression of ALDH1 and DBR2. The contents of artemisinin were remarkably increased in the transgenic plants of *A. annua* with DBR2 overexpression [43]. Blue light inhibited the expression of ESC and SQS, which catalyzed FPP to 8-epi-cedrol and squalene, respectively. A study showed that suppressing the expression of SQS significantly increased the artemisinin content in transgenic *A. annua* [44]. Hence, light, especially blue light, not only promoted the expression of artemisinin synthetase, but it could have also inhibited the expression of synthetases in the branching pathway, which allowed more metabolites to flow to artemisinin. 

### 2.7. Validation of Differential Expression via Quantitative Real-Time PCR (qRT-PCR)

**Figure 6:**
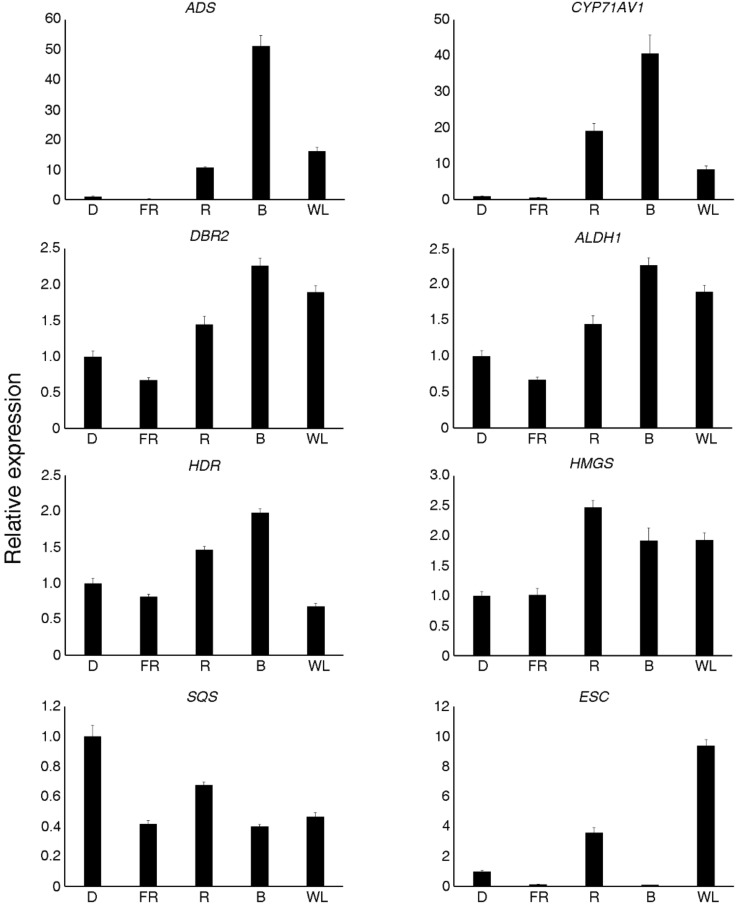
Validation of RNA sequencing results by real-time quantitative PCR. D—darkness; FR—far red light; WL—white light; R—red light; B—blue light. ADS—amorpha-4,11-diene synthase; CYP71AV1—amorphadiene-12-hydroxylase; DBR2—artemisinic aldehyde Δ11(13) reductase; ALDH1—aldehyde dehydrogenase 1; HDR—hydroxy-2-methyl-2-(*E*)-butenyl 4-diphosphate reductase; HMGS—3-hydroxy-3-methyl-glutaryl coenzyme A synthase; SQS—squalene synthase; ECS—epi-cedrol synthase. The relative expression levels were normalized to that of the *actin* control. Data represent means ± standard deviation of biological triplicates.

The levels of DEGs were validated using qRT-PCR and eight selected genes. The qRT-PCR expression profile showed a positive correlation with the transcriptome data (Figure 6 and Table S1). 

### 2.8. Identification of Co-Expressed TFs and Artemisinin Biosynthesis Genes

**Figure 7:**
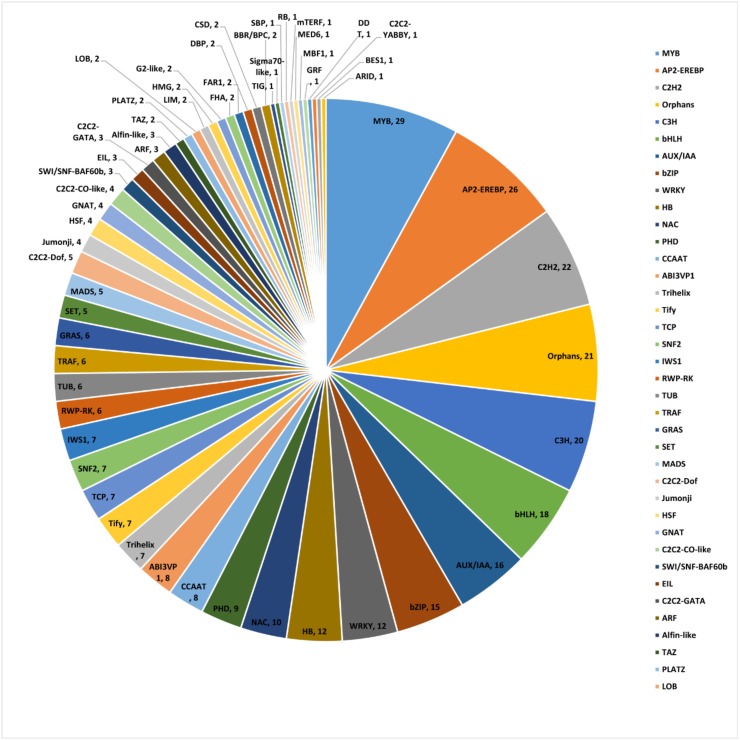
Distribution of transcription factor (TF) families in the co-expression results. There were 365 TFs that were co-expressed with TRINITY_DN75917_c2_g1 (ADS) and TRINITY_DN83022_c1_g1 (CYP71AV1) (r > 0.95).

For more information about the regulation of the genes that were involved in the artemisinin biosynthesis, transcription factors were identified using the iTAK software. We identified a total of 5106 TFs. Then, the co-expression analysis was carried out to obtain the specific TFs that might have regulated the artemisinin biosynthesis gene expression (Table S2), which revealed that 365 TFs were co-expressed with TRINITY_DN75917_c2_g1 (ADS) and TRINITY_DN83022_c1_g1 (CYP71AV1) (|r| > 0.95). Within these 365 TFs, 29 were MYB TFs, 18 were bHLH TFs, 15 were bZIP TFs, and 12 were WRKY TFs (Figure 7). These TF families had been reported to play important roles in regulating artemisinin biosynthesis gene expression. 

**Figure 8:**
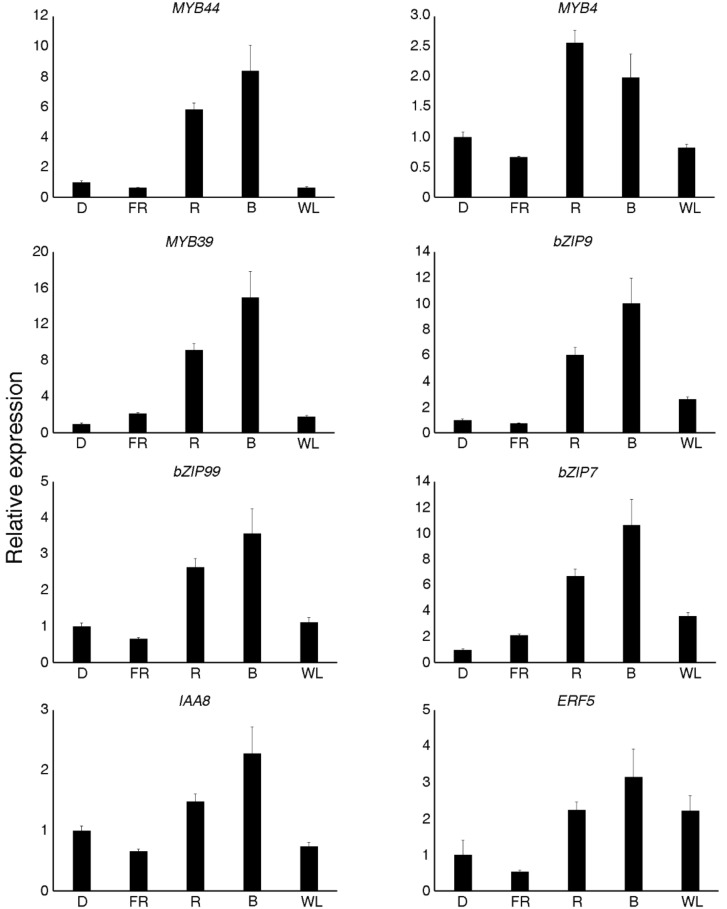
Quantitative real time PCR results obtained for selected transcription factors. D—darkness; FR—far red light; WL—white light; R—red light; B—blue light. The relative expression levels were normalized to that of the *actin* control. Data represent means ± standard deviation of biological triplicates.

In order to validate the TF gene expression results, eight TFs that were co-expressed with ADS and CYP71AV1 were selected for qRT-PCR analysis (Figure 8), and the qRT-PCR expression profile showed a positive correlation with the transcriptome data. Three MYB TFs, three bZIP TFs, one IAA TF, and one ERF TF showed the highest expression under blue light, followed by red light, except MYB4, which showed the highest expression under red light.

In the plants, the synthesis and accumulation of secondary metabolites were under strict control from the TFs. Transcriptional regulation was a complex network, as a single TF might have regulated the expression of many genes that were involved in a specific metabolic pathway, and many TFs regulated the expression of a specific gene [25]. Several TF families, including WRKY, AP2/ERF, bHLH, and bZIP, were involved in the regulation of artemisinin biosynthesis. Most of these TFs were involved in hormone signals, but the TF that was regulated by light was still unknown. To explore which TFs might have been regulated by light, a co-expression analysis was carried out. The results revealed that MYB TFs were the most frequent TFs in the process, and that their expression was induced by light. In addition, 18 bHLH TFs and 15 bZIP TFs were found (Figure 7). In the light signal transduction pathway, the well-known bZIP TF HY5 was the central point of the pathway [45,46]. The phytochrome interacting factors (PIFs) belonging to the bHLH family were also very important light signal TFs, which regulated many of the development processes [47]. Twelve WRKY TFs were also found, and the WRKY TFs were usually related with light stress response and were induced by light when plants transitioned from heterotrophic to autotrophic growth [48]. Many TFs were found by co-expression analysis and were induced by light (Figure 8), and thus might have been involved in the light signal pathway, interacting with HY5 or PIFs. Overall, the results that were presented here provided candidate TFs for the transcriptional regulation of artemisinin by light, which was of great significance for further in-depth studies regarding artemisinin synthesis regulation.

## 3. Materials and Methods

### 3.1. Plant Material and Growth Conditions

The *Artemisia annua* that was used in this study was a wild type, which we named ‘Haiqing 1’, was obtained from Haikou, Hainan, China. The *Artemisia annua* seeds were sown to the soil for four weeks under white light at 25 °C. The photoperiod in the growth chamber was 16/8 h (light/dark) (16 h light and 8 h darkness per day). Then, the seedlings were transferred to four continuous light treatments for two days before harvesting. After different light treatments, we collected the aboveground parts of 10 seedlings randomly, as a sample approximately 0.8 g. Then, the sample was grinded into powder and put into liquid nitrogen. The content of artemisinin was detected using 0.1 g of the powder, and the other 0.1 g was used to extract RNA for sequencing. Light-emitting diode (LED) red light (670 nm), LED blue light (470 nm), and LED far-red light (735 nm). The light intensity was 50 ± 5 µmol/ m^2^ s in all of the treatments. Each treatment comprised three biological replicates (10 seedlings per repeat).

### 3.2. Chlorophyll Measurement

The chlorophyll measurement was conducted as previously described [49]. Chlorophyll content was expressed as 7.18 × OD663 + 17.32 × OD646 per 20 seedlings. All of the experiments were performed in triplicate.

### 3.3. Transcriptome Sequencing

The process was described previously [50], the plant total RNA was isolated using an RNA Extraction Kit (Tiangen, Beijing, China) according to the manufacturer’s instructions. To build the sequencing library, two micrograms of total RNA, with 28S/18S RNA ratio ≥1.8 were used by the NEBNext^®^ Ultra RNA Library Prep Kit (New England Biolabs Inc., Ipswich, MA, USA). The sequencing library quality was determined with Agilent 2100 Bioanalyzer, which had a minimum RNA integrity number (RIN) value of 7 and 250–300 bp insertion elements. The RNA sequencing was performed using the Illumina Xten sequencing system (Illumina Inc., San Diego, CA, USA).

### 3.4. De Novo Assembly

In order to obtain high quality clean reads, the adapter sequences, low-quality sequences with N (undetermined bases) percentage over 10%, and the sequences containing more than 50% bases with *q*-value ≤5 and shorter than 35 bases, were removed. Clean reads were assembled into contigs de novo by the Trinity software with the min_kmer_cov set to 2 and all of the other parameters set to default values [51]. The program TransDecoder was used to predict the complete open reading frames (ORFs) of the unigenes (https://transdecoder.github.io/).

### 3.5. Functional Annotation 

For the functional annotation, the resulting contigs were blasted against the National Center for Biotechnology Information (NCBI, http://www.ncbi.nlm.nih.gov) non-redundant protein database (NR) and nucleotide sequences (NT) (*e*-value = 1e^−5^), the Pfam database hmmscan (*e*-value = 0.01) (http://pfam.sanger.ac.uk/), the Swiss-Prot (*e*-value = 1e^−5^) (http://www.ebi.ac.uk/uniprot/), euKaryotic Ortholog Groups (KOG) (*e*-value = 1e^−5^) (http://www.ncbi.nlm.nih.gov/COG/), and the Kyoto Encyclopedia of Genes and Genomes (KEGG), using the KEGG Automatic Annotation Server (*e*-value = 1e^−10^) (http://www.genome.jp/kegg/). Subsequently, the gene ontology (GO) annotation was performed using the Blast2GO v2.5 (*e*-value =1e^−6^) (http://www.geneontology.org/) [52].

### 3.6. Transcription Factor Identification and Co-Expression Analysis

The transcription factor families were identified using the iTAKsoftware. In order to select the possible TFs regulating artemisinin biosynthesis, two highly expressed genes—TRINITY_DN75917_c2_g1 (*ADS*) and TRINITY_DN83022_c1_g1 (*CYP*71AV1)—were used in co-expression analysis, with a default value 0.6 [53]. The paired genes showing a Pearson correlation coefficient (r) greater than 0.95 were considered as significantly co-expressed and were selected to build a co-expression network, using the Perl script [50]. The data correlation and visualization were performed using Cytoscape v3.4.10 (National Institute of General Medical Sciences, Bethesda, MD, U.S.) [54]. 

### 3.7. Quantitative Real-Time PCR (qRT-PCR) Analysis of Gene Expression

The plant total RNA was isolated using an RNA Extraction Kit (Tiangen) and the first-strand cDNA was synthesized from 2 mg of RNA by reverse transcriptase (Invitrogen, Carlsbad, CA, USA), and then diluted (1:100) for use in qRT-PCR, with SYBR Premix ExTaq Mix (Takara, Dalian, Liaoning, China) in a total volume of 15 mL. The reactions were performed in a LightCycler 480 thermal cycler (Roche, Basel, Switzerland), following the manufacturer’s instructions. Three biological replicates were performed for each sample, and the expression level was normalized to that of *actin*, which was used as the control gene. 

### 3.8. Liquid Chromatography–Mass Spectrometry (LC–MS) Analysis of Secondary Metabolites

Of the sample powder, 0.1 g was extracted overnight at 4 °C with 1.0 mL pure methanol (or 70% aqueous methanol) containing 0.1 mg·L^−1^ lidocaine. The lipid-solubility extracts were absorbed and 0.4 mL of each extract was mixed and filtrated (SCAA-104, 0.22 µm pore size) before LC–MS analysis. Artemisinin and artemisinic acid reference substances were accurately weighed (0.001 g) and used to obtain a stock solution (1 mg/mL), which was dissolved by adding the corresponding volume of methanol. The stock solutions of artemisinin and artemisinic acid standards were diluted to 100 µg/mL, 1600 ng/mL, 800 ng/mL, 400 ng/mL, 200 ng/mL, 100 ng/mL, 50 ng/mL, 25 ng/mL, and 10 ng/mL. The standard curves were determined as Y = 600.39X + 6012.6, and the linear range was 10–800 ng/mL.

### 3.9. High-Performance Liquid Chromatography (HPLC) Conditions

The analytical conditions were as follows: column was Agilent Elipse Plus C18 column (Santa Clara, CA, USA., pore size 1.8 µm, length 2.1 × 50 mm); solvent system was water (0.1% formic acid), acetonitrile (0.1% formic acid); gradient program was 100:0 *v*/*v* at 0 min, 5:95 *v*/*v*, at 20.0 min, 5:95 *v*/*v* at 22.0 min, 95:5 *v*/*v* at 22.1 min, and 95:5 *v*/*v* at 28 min; flow rate was 0.2 mL·min^−1^; temperature was 40 °C; injection volume was 1 µL. The effluent was alternatively connected to an electrospray ionization (ESI)-triple quadrupole-linear ion trap (Q-TRAP)-MS or ESI-QqTOF-MS.

## 4. Conclusions

Using LC–MS analysis, the present study revealed that red and blue light promoted artemisinin and artemisinic acid content in *A. annua* seedlings. Additionally, GO annotations revealed that the up-regulated genes under red and blue light treatments were closely related to photosynthesis, and the heatmap and qRT-PCR analyses revealed that red and blue light led to the up-regulation of many artemisinin biosynthesis genes and inhibited the expression of synthetases in the branching pathway. The co-expression was analysis carried out to reveal the TFs that might have regulated the artemisinin biosynthesis gene expression, and the qRT-PCR analysis confirmed that the expression of MYB39 and bZIP9, among others, were induced by red and blue light. Overall, the results that have been presented here revealed that red and blue light promoted the accumulation of artemisinin and artemisinic acid in *A. annua.*

## Figures and Tables

**Table 1:** Summary of the *Artemisia annua* transcriptome data obtained by Illumina sequencing.

Sample	Clean Reads (n)	Clean Bases (n)	Q20 (%)	Q30 (%)	GC Content (%)
D1	41,315,654	39,620,944	97.63	93.66	42.76
D2	44,4941,98	42,460,928	97.32	92.96	42.92
D3	45,997,162	44,196,856	97.62	93.63	42.85
FR1	44,583,758	42,993,870	97.60	93.58	42.92
FR2	46,616,436	44,924,628	97.65	93.80	42.92
FR3	53,563,708	51,433,966	97.16	92.79	42.93
WL1	48,061,222	46,239,326	97.56	93.62	42.67
WL2	41,232,122	39,771,772	97.63	93.77	42.75
WL3	51,498,242	49,412,744	97.68	93.87	42.82
R1	46,278,940	44,459,758	96.90	92.12	42.62
R2	50,660,678	48,157,446	97.32	93.01	42.55
R3	50,543,012	47,291,920	97.21	92.90	42.61
B1	63,937,458	61,502,154	97.95	94.64	42.50
B2	60,062,102	57,833,940	97.91	94.54	42.52
B3	55,204,498	52,916,964	97.59	93.60	42.51

D—darkness; FR—far-red light; WL—white light; R—red light; B—blue light. Numbers correspond to three biological duplicates. Q20 and Q30: the base value of Phred is greater than 20 and 30, respectively, accounting for the total base percentage.

**Table 2:** Unigenes annotated per database.

Database	Number of Unigenes	Percentage (%)
NR	90,049	33.11
NT	67,311	24.75
KEGG	37,459	13.77
Swiss-Prot	83,506	30.70
PFAM	85,702	31.51
GO	87,236	32.08
KOG	36,681	13.48
Annotated in all of the databases	13,326	4.90
Annotated in at least one database	136,365	50.14
Total Unigenes	271,932	100.00

NCBI—National Center for Biotechnology Information; NCBI NR—NCBI non-redundant database; NCBI NT—NCBI nucleotide sequences; KEGG—Kyoto Encyclopedia of Genes and Genomes; SwissProt—a manually annotated and reviewed protein sequence database; PFAM—Protein family; GO—Gene Ontology; KOG—euKaryotic Ortholog Groups.

## Supplementary Materials

The chlorophyll content under different light treatments, Figure S2: Functional classification and pathway assignment of assembled unigenes by KEGG, Figure S3: The KOG annotation for all assembled unigenes. Table S1: List of primers used in qRT-PCR, Table S2: List of co-expressed transcript factors.
