# Supplementary figures and images for: Red and Blue Light Promote the Accumulation of Artemisinin in Artemisia annua L
Source: Molecules. 2018 May 31;23(6):1329. doi: 10.3390/molecules23061329 (PMC6100300; doi:10.3390/molecules23061329)

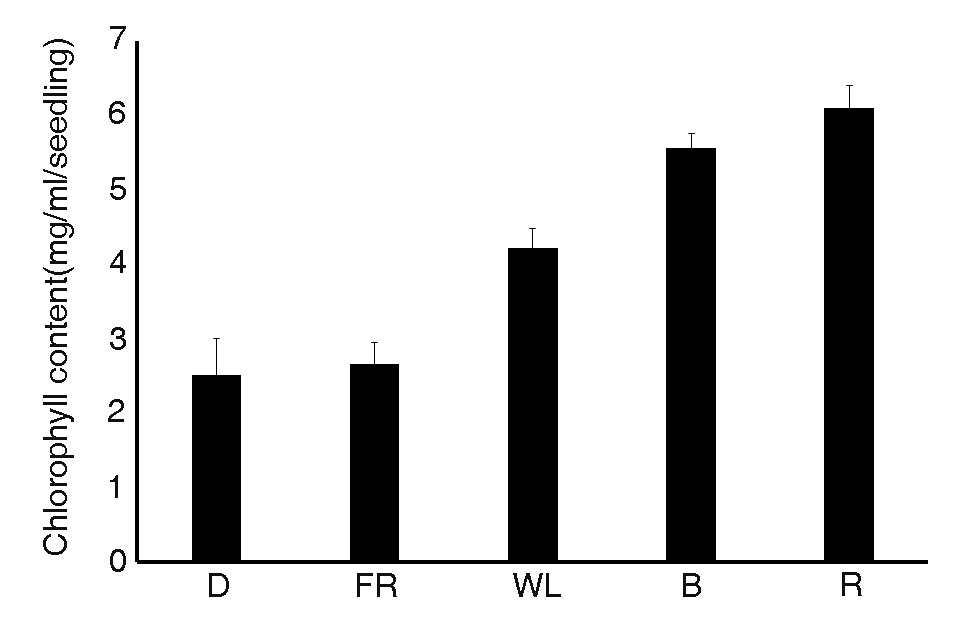

Supplement: Supplementary file 1 [file molecules-23-01329-s001.zip › Supplementary data/Figure S1.tif]

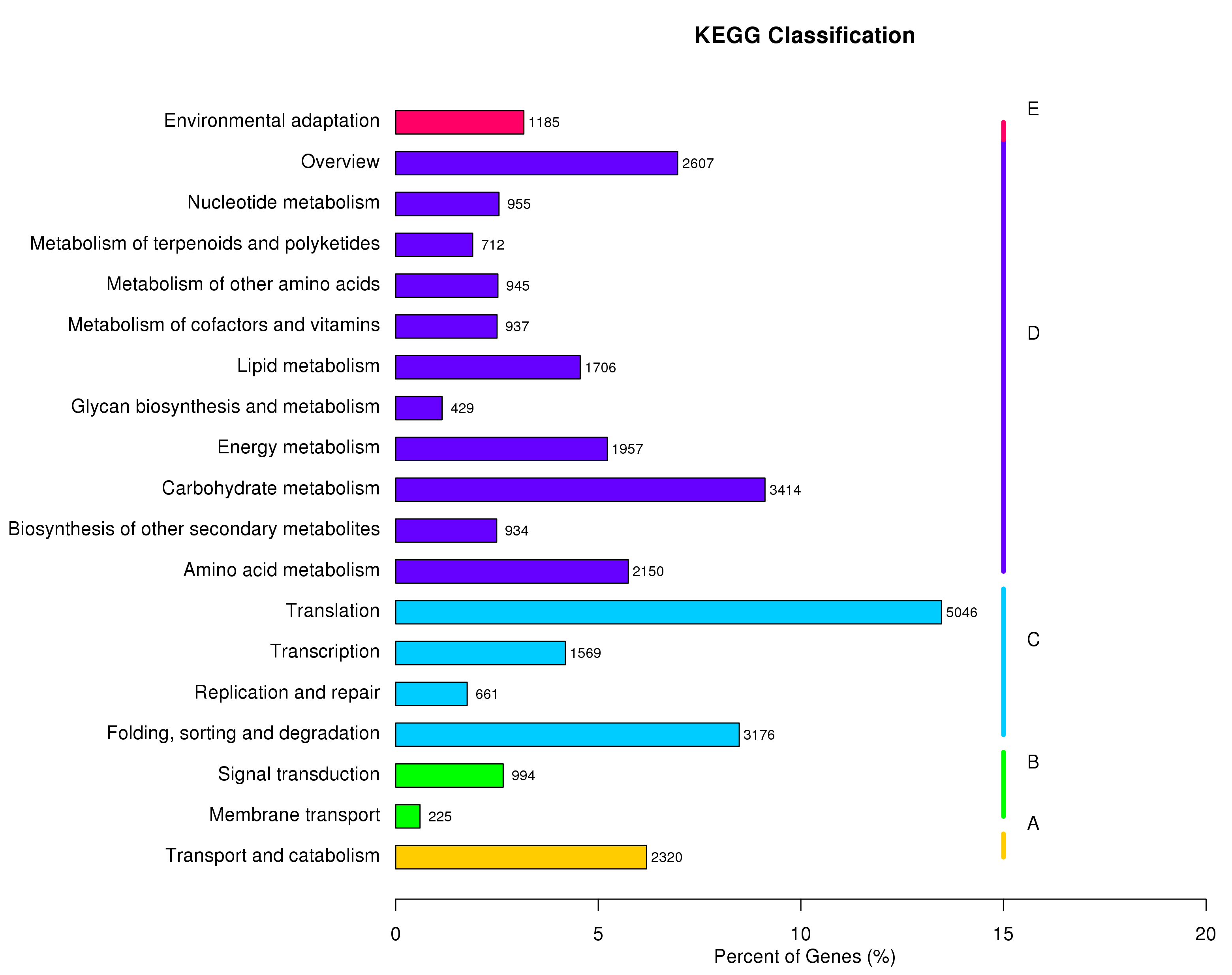

Supplement: Supplementary file 1 [file molecules-23-01329-s001.zip › Supplementary data/Figure S2.tif]

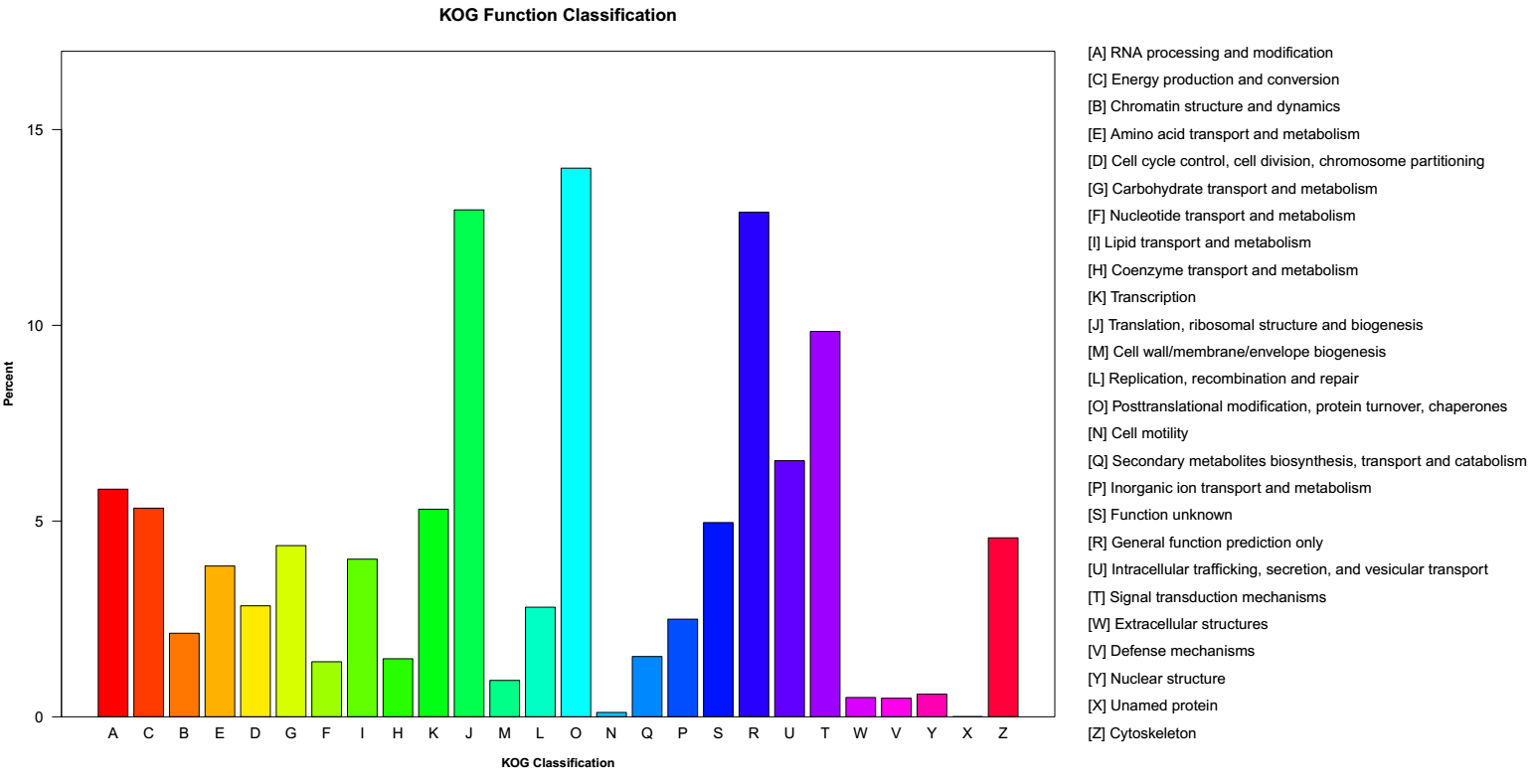

Supplement: Supplementary file 1 [file molecules-23-01329-s001.zip › Supplementary data/Figure S3.tif]
